# Comparison of Microleakage of MTA and CEM Cement Apical Plugs in Three Different Media

**DOI:** 10.7508/iej.2016.03.010

**Published:** 2016-05-01

**Authors:** Fatemeh Ayatollahi, Mahdi Tabrizizadeh, Milad Hazeri Baqdad Abad, Reza Ayatollahi, Fatemeh Zarebidoki

**Affiliations:** a *Department of Endodontics, Dental School, Shahid Sadoughi University of Medical Sciences, Yazd, Iran;*; b * Dental Student, Dental School, Shahid Sadoughi University of Medical Sciences, Yazd, Iran*

**Keywords:** Calcium-Enriched Mixture Cement, Microleakage, Mineral Trioxide Aggregate

## Abstract

**Introduction:**

Microleakage is of the causes of endodontic treatment failure. The aim of this *in vitro *study, was to compare the microleakage of mineral trioxide aggregate (MTA) and calcium-enriched mixture (CEM) cement apical plugs in three environments.

**Methods and Materials:**

A total of 130 human extracted single rooted teeth were collected. After decoronation, preparation of the root canal space and simulation of open apices, 5 teeth were selected as positive and negative control groups and the rest of the samples were randomly divided into two groups (MTA plug and CEM cement plug) and each group was divided into 3 subgroups (dry, contaminated with saliva and contaminated with blood). In each group apical plug was placed into the canal. After full setting of the apical plug, microleakage of the samples were evaluated using fluid filtration method and the data were analyzed using two-way analysis of variance.

**Results:**

In dry and saliva contaminated environments, the leakage of MTA samples were 40.906±2.081 and 39.608±2.081, respectively which was significantly more than that of CEM cement samples (26.977±2.081 and 27.000±2.081, respectively). However, in blood-contaminated environments, the amount of leakage in MTA group (21.640±2.081) was significantly lower than CEM cement group (44.358±2.081).

**Conclusion:**

According to the results of this study, in dry and saliva-contaminated conditions CEM cement provides significantly better seal in comparison to MTA

## Introduction

Pulp necrosis can occur before maturation of root apex. This phenomenon lead to ceasing of odontogenesis and root development [1-4]. Root canal treatment of an immature tooth is always difficult, due to the lack of supporting point for gutta-percha compaction and it can lead to extrusion of filling materials from the apical foramen and periapical damage [2, 3].

For many years, apexification (induction of a calcific barrier in an open-apex necrotic tooth) was considered as one of the most common methods for treating nonvital immature teeth. This technique involves removal of the necrotic tissue, debridement of the canal and placement of an antimicrobial material such as calcium hydroxide for induction of a calcific apical barrier [5, 6]. But this method has its own problems such as requiring numeral treatment sessions and its dependence on the cooperation of patient. In addition, the long-time use of calcium hydroxide would increase the probability of tooth fracture [1, 7].

Pulp regeneration is another technique for treatment of immature necrotic teeth. The purpose of this technique is to control intracanal infection and induction of connective tissue and promotion of normal root development. Beside promising advantages, this method needs more investigation for routine clinical application [7, 8].

According to many studies, the use of apical plug is easier and more effective than apexification method. In this method, an apical plug is used as an apical barrier and a supporting point for the gutta-percha compaction [1, 6, 9].

Calcium hydroxide, tricalcium phosphate, absorbable ceramic, dentin chips, mineral trioxide aggregate (MTA) and calcium-enriched mixture (CEM) cement are some of the materials used in the apical plug method [9, 10].

MTA consists of a hydrophilic powder that after hydration will set hard and become a tough mass 24 h after mixing. The primary pH of mixed MTA is 10.2 and reaches 12.5 after setting. MTA has high sealing ability, proper marginal fit and good tissue compatibility. In addition, the solubility and compressive strength of MTA is adequate. The disadvantages of MTA include high price, long setting time, potential of dentin discoloration and poor handling properties [11-13].

CEM cement on the other hand consists of calcium compounds such as calcium oxide, calcium carbonate, calcium silicate, calcium sulfate, calcium hydroxide and calcium chloride. The setting time and film thickness of CEM cement are lower than MTA. The antibacterial effect of CEM cement is more than MTA and comparable to that of calcium hydroxide [11]. According to some studies, the sealing ability of CEM cement is better than MTA, while based on other studies the difference in microleakage between these two materials is not statistically significant [8, 11, 14-16].

Microleakage is one of the important reasons for the failure of endodontic treatment [17]. A study by Toronto group indicated that 88% of endodontic treatment failures are due to apical microleakage [18]. Blood and saliva contamination during root canal obturation is one of the factors that influences the microleakage [19]. Several studies have approved that various dental materials in dry condition have better seal than wet environment [20, 21]. However, the study by Kuhre and Kessler [22] did not report any significant difference between dry and wet environments.

However, the presence of blood does not have any significant effect on microleakage of the root end cavities filled with MTA, but microleakage of MTA in the cavities contaminated with saliva is significantly higher than CEM cement [23, 24].

Studies on the effect of blood and saliva contamination on microleakage of CEM cement are limited. In this *in vitro *study, the microleakage of apical plug of MTA and CEM cement were compared in three dry, blood- and saliva-contaminated environments.

## Materials and Methods

The study protocol was approved by research department of Shahid Sadoughi University of Medical Sciences (Grant No.: 702). A total of 130 extracted human single canal teeth were chosen that were without severe caries, root fracture and dilacerations, no large coronal restoration, lack of internal and external absorption and canal calcification.

After 1 h maintenance in 5.25% sodium hypochlorite solution (for disinfection and elimination of soft tissue remnants), the teeth were kept in normal saline. At the beginning of the study, a diamond disc (Tizkavan, Tehran, Iran) was used to cut off the crowns to keep 13 mm of the root length. A #15 K-file (Dentsply Maillefer, Ballaigues, Switzerland) was used to determine the working length.

Preflaring of the coronal part of canal was performed using Gates-Glidden drills (Dentsply Maillefer, Ballaigues, Switzerland), the apical region was cleaned using K-Files up to size #40 and the root canal was flared to #80. The canals were irrigated with 2.25% sodium hypochlorite during instrumentation. The next step involved the orthograde use of #1 to 4 Peeso Reamer drills (Dentsply, Maillefer, Tulsa, OK,USA) to create an apical foramen with 1.3 mm width for simulation of open apex conditions [25].

In order to remove the smear layer, canals were filled with 1 mL of 17% ethylenediaminetetraacetic acid (EDTA) (Ariadent, Tehran, Iran) for 3 min and then, they were washed with 5mL of normal saline and dried with paper cones.

Five teeth were obturated with gutta-percha (Diadent, Chongju, Korea) without sealer and were considered as the positive control samples. Moreover, 5 teeth were covered with sticky wax (Kerr, Berlin, Germany) in the apical area and their surfaces were coated with two layers of nail polish, to be put in negative control group. Other roots were randomly divided into two groups in which the apical plugs were placed using either MTA or CEM cement. Each group were then divided into 3 subgroups: dry, blood- and saliva-contaminated canal.

*Group 1A*: In this group after drying the canals, White MTA (Angelus, Londrina, PR, Brazil) was mixed according to the manufacturer's instructions and was placed inside the canal using MTA carrier (Dentsply Maillefer, Ballaigues, Switzerland) and it was condensed with a hand plugger (Dentsply Maillefer, Ballaigues, Switzerland). A 5-mm thick apical plug was placed in each sample. After evaluation of the thickness and density of the apical plug with radiography, a wet paper point was put in the canal and the access cavity was filled by temporary restoration (Coltosol, Ariadent, Tehran, Iran). Samples were kept in the incubator at 37^°^C and 100% humidity for 24 h. Then, the temporary restoration was removed and complete setting of the MTA was checked. Canal irrigation was done and the canal space was filled with gutta-percha (Diadent, Chongju, Korea) and AH 26 sealer (Dentsply, DeTrey, Konstanz, Germany) using lateral condensation technique.

*Group 1B*: Canal was filled with human blood (taken from volunteers) and then additional blood was aspirated until only the walls remained contaminated with blood at the end. The other stages were the same as group 1A. 

*Group 1C*: Canals were filled with fresh human saliva so that only the walls remained contaminated with saliva. The other stages were the same as previous groups.

**Table 1:** Mean (SD) of microleakage (10⁻⁴×µL/min/CmH_2_O)

	**Dry**	**Blood **		**Saliva **
**MTA**	40.906 (2.081)		21.640 (2.081)	39.608 (2.081)
**CEM cement**	26.977 (2.081)		44.358 (2.081)	27.000 (2.081)
***P*** **-value**	0.000		0.000	0.000

In the next three sub-groups (2A, 2B and 2C), all the procedures were the same as the MTA group and CEM cement (Bionique Dent, Tehran, Iran) was used instead for the apical plug insertion. 

Samples were incubated for 24 h in 100% humidity and 37^°^C. After this period of time, the amount of microleakage was evaluated using fluid filtration method as described by Moradi *et al. *[26]. The surfaces of sample were coated with two layers of parafilm except for the apical 2 mm. The apical part of samples were connected to a plastic tube with cyanoacrylate glue. The other side of the tube was connected to a plastic three valve adaptor which was connected to a standard glass capillary tube. All spaces at the apical side of samples (plastic tubes, pipettes, syringes) were filled with distilled water. Water was sucked using syringe and bubbles were created. A pressure of 0.5 ATM was created at the end of capillary tube to displace air bubble. The movement of the air bubble was observed and the volume of the fluid transport was measured by this observation [26].

Microleakage of each sample was measured for 3 times and the average value was considered as the final value of the microleakage. Finally, the data were analyzed using the two-way analysis of variance .The level of significance was set at 0.05. In order to do this process, the SPSS software (SPSS version 18.0, SPSS, Chicago, IL, USA) was utilized.

## Results

According to the results of this study, the positive control group showed the greatest amount of the bubble movement, while in the negative control group, there was no movement of the bubbles. Mean and standard deviation of bubble microleakage are summarized in Table 1.

In dry and saliva-contaminated environments the microleakage of MTA is significantly more than CEM cement (*P*<0.001). However, in blood-contaminated environment, the amount of microleakage in MTA group was significantly lower than CEM cement group (*P*<0.001). 

## Discussion

The science of endodontics has been always looking for the better ways for treatment of the immature necrotic teeth. Today, the apical plug method is regarded as the golden standard due to its advantages such as short treatment time, less need for patient’s cooperation and satisfactory results [7].

The sealing ability of the apical plug can be affected by the moisture such as blood and saliva. Therefore, in this study we compared the sealing ability of two materials in different conditions [12, 27, 28].

There are a variety of methods to determine the sealing ability of materials, including color penetration, bacterial leakage, protein penetration, glucose penetration, electro-chemical methods, electromagnetic isotope and fluid filtration. In the present study, the fluid filtration method was used, due to being more sensitive than dye penetration method. In addition, in this way, the microleakage can be measured without destroying the samples [24, 29, 30]. In the fluid filtration method, sealing ability was measured by the movement of an air bubble in a capillary tube under pressure. The investigation of the amount of microleakage using fluid filtration method is a quantitative method and it is more sensitive compared to color penetration method. In this method the samples are not destroyed and the evaluation of the amount of microleakage over time is possible. On the other hand, the lack of tracer molecules eliminates the possibility of errors [25, 29, 31-35].

In a study by Torabinejad *et al. *[23], the sealing ability of amalgam, MTA, IRM and Super EBA was investigated as root end filling materials in blood-contaminated canals using dye penetration method. They asserted that the sealing ability of MTA in blood contaminated condition is significantly more than dryness. The results of some other studies witch have shown that in the dry canal, CEM cement has less microleakage than MTA, confirmed the results of the present study [16, 24, 36].

In another study, Hasheminia *et al*. [24] evaluated the effect of blood and saliva contamination on the microleakage of root end fillings with MTA and CEM cement. They reported that in the saliva contamination condition, the microleakage of CEM cement was significantly less than MTA. On the other hand, the sealing ability of CEM cement was better than MTA in dry environment, although this difference was not significant. These results are consistent with of the results of the present study.

As CEM is a water-based cement, moisture may positively impact its chemical reaction and setting. Saliva by moistening the walls of the canal will increase the concurrence of cement poison with irregularities of the canal and it caused the penetration in the dentinal tubules [24, 37]. The material in the water increases the pH values by releasing large amounts of phosphate, calcium and hydroxyl ions [38]. In addition, positioning of a cement with hydroxide apatite production capability also resulted in further compliance with the walls of the canal and thereby better seal [29, 39]. 

## Conclusion

According to the results of this study, CEM cement in dry and saliva-contaminated canals provides significantly better seal in comparison with MTA.
